# Comparative Efficacy of Botulinum Toxin in Salivary Glands vs. Oromotor Therapy in the Management of Sialorrhea in Cerebral Palsy Impact on Sleep Quality

**DOI:** 10.3389/fresc.2022.875235

**Published:** 2022-06-16

**Authors:** Juan Francisco Marquez-Vazquez, María Elena Arellano-Saldaña, Karla Nayeli Rojas-Martinez, Paul Carrillo-Mora

**Affiliations:** ^1^Master and Doctorate Division, Instituto Nacional de Rehabilitación LGII, Tlalpan, México; ^2^Pediatric Rehabilitation Service, Instituto Nacional de Rehabilitación LGII, Tlalpan, México; ^3^Neuroscience Research Division, Instituto Nacional de Rehabilitación LGII, Tlalpan, México

**Keywords:** cerebral palsy, botulinum toxin type A, sleep disorders, sialorrhea, oromotor assessment

## Abstract

**Aims:**

The aim of this study was to compare the effects of intraglandular abobotuliniumtoxinA application and oromotor therapy in the management of sialorrhea in patients with cerebral palsy and its effect on sleep quality.

**Methods:**

A comparative study (*n* = 134), mean age 7.1 years (± 3.9 years) was performed in pediatric patients, between the efficacy of abobotuliniumtoxinA in salivary glands and oromotor therapy (JT), with a control group receiving exclusive oromotor therapy (EOMT). Demographic variables, as well as Gross Motor Function Classification System (GMFCS), Drooling Severity and Frequency Scale (DSFS), Sleep Disturbance Scale for Children (SDSC) and Eating and Drinking Ability Classification System (EDACS) were analyzed in 134 patients considering two measurements 6 months apart. Statistical analysis was developed between both groups.

**Results:**

The greatest improvement in safety and efficacy of swallowing were those in the JT group with initial levels of EDACS IV and V. Both therapies result in favorable changes of all subscales means of SDSC, with joint therapy showing the greater benefit (*p* = 0.003) over EOMT (*p* = 0.06), especially for Sleep Breathing Disorders and Disorders of initiating and maintaining sleep (*p* < 0.01 vs. *p* = 0.07). No major adverse effects were found, only those expected from the application of the toxin, such as pain, mild, and transient local inflammation.

**Interpretation:**

A correlation between frequency and intensity of sialorrhea, with the frequency of sleep disorders and dysphagia was found. Conventional EOMT proved to be useful, improving the safety and efficacy of swallowing, sialorrhea and sleep disorder, however it can be enhanced with the application of abobotuliniumtoxinA.

## What this Paper Adds

- There is a correlation between: severity of the sialorrhea/sleep disorders and dysphagia.
- AbobotuliniumtoxinA plus oromotor therapy improves safety and efficacy of swallowing.
- Enhanced orofacial therapy with abobotuliniumtoxinA is helpful in treating sleep disorders.
- Systematic screening of sleep disorders, and swallowing effectiveness/safety is recommended.

## Introduction

The clinical diagnosis of cerebral palsy (CP) defines a group of permanent disorders in the development of movement and posture, causing functional limitation, attributed to non-progressive disturbances that occurred during fetal development and infant brain (1). CP is associated with many comorbidities, including vision impairment, intellectual disability, respiratory conditions, epilepsy, and sleep disturbance. Current research suggests that between 30 and 60% of children with CP have sleep problems (2). Sialorrhea is a common concern among children with neurological disorders, prevalence of moderate or severe sialorrhea in this population is estimated at between 10 and 37% (3). Sialorrhea results from failed clearance and removal of saliva from the oral cavity, most often associated with impaired swallowing (4). Copious sialorrhea can cause psychosocial problems for the family and the developing child, while in severe cases, children can aspirate their saliva and develop recurrent pneumonia (5). The application of botulinum toxin A (abobotuliniumtoxinA) has been shown to be a safe and effective option to treat children with moderate and severe sialorrhea, improving quality of life as well as raising the patient's self-esteem, achieving better acceptance and facilitating care of these patients (4, 6). In our developing country context, a possible limitation to the use of treatments such as botulinum toxin is its cost, so comparing and knowing its efficacy with other more accessible techniques is of great clinical relevance. The aim of this study is to compare the effects of intraglandular abobotuliniumtoxinA application and oromotor therapy in the management of sialorrhea in patients with CP, on sleep quality, and other clinical variables. Despite the fact that oromotor therapy and the application of abobobotuliniumA toxin have been used as part of the management of sialorrhea (7), we found no publications comparing the efficacy of these two interventions.

## Methods

### Study Design

Comparative, longitudinal, descriptive and analytical clinical study.

### Population

The study was performed in patients treated at the pediatric rehabilitation service of the National Rehabilitation Institute of Mexico (INR-LGII), where the functional changes were analyzed comparing the application of abobotuliniumtoxinA in salivary glands and oromotor therapy, with a control group receiving exclusive oromotor therapy, and its association with clinical variables such as severity of sialorrhea, presence of swallowing disorders, sleep disorders, among others. We worked with a total of 134 patients divided by convenience (not randomized) into two groups that were matched by age and sex, group A, attended with exclusive oromotor therapy with 68 patients and group B attended with oral-motor therapy and application of abobotuliniumtoxinA to salivary glands with 66 individuals.

### Inclusion Criteria

Patients of any gender, between 3 and 17 years old at the beginning of the study, whose reason for consultation was CP and whose parents voluntarily agreed to be part of the study by signing an informed consent. Patients with severe sialorrhea according to the Drooling severity and frequency Scale (DSFS) (8) were included.

### Elimination Criteria

Patients who do not complete all the tests or those who expressly wished to exit the protocol.

### Exclusion Criteria

Patients in whom the cause of sialorrea was not clearly demonstrated to be associated with CP. Patients who do not accept their entry to the protocol.

### Procedure

Five units of abobotuliniumtoxinA were applied to the patients with moderate sialorrhea and 10 units to those with severe sialorrhea. The dilution used was 100 units in 1 ml of 0.9% saline solution. The technique used for injection was as follows: The glands were located by ultrasound guidance with linear transducer. A 1 ml syringe was used for the application of the solution (5 or 10 U of botulinum toxin A), injected into each submandibular, parotid gland, or both according to severity of sialorrhea. Children were observed at least for 1 h after procedure to observe if any side or adverse effects were shown.

### Measurements

Questionnaires were carried out to determine demographic variables, Gross Motor Function Classification System (GMFCS), clinical classification of the intensity and frequency of the sialorrhea, Sleep disturbance Scale for Children (SDSC), and EDACS (Eating and Drinking Ability Classification System). All of them were carried out in two measurements 6 months apart.

### Ethical Considerations

All parents or tutors signed an informed consent letter and the protocol was accepted by the research and bioethics committee of the INR-LGII according to current regulations.

### Statistical Analysis

Statistical analysis was performed with SPSS version 24 using non-parametric Wilcoxon statistical contrasts and marginal homogeneity for related samples. For the initial comparison of the groups Student's *t*-test was performed for independent data in quantitative variables and chi squared for qualitative variables, finally logistic regression models were established to determine statistical significance.

## Results

The sample was 134 patients (46 female and 88 male), all of them completed the study. The mean general age was 7.1 years (± 3.9 years). The patients were divided in two groups. Group A (*n* = 68) belongs to those patients who received orofacial therapy (oral cavity stimulation, swallowing exercises, sensitization to textures, etc.), and group B (*n* = 66) those who received orofacial therapy in the same way as group A, plus the application of abobotuliniumtoxinA to salivary glands according to the INR's protocols. In both groups the male female ratio was 2:1. Descriptive data result is shown in Table 1. Regarding the predominant motor disorder, the most frequent type was the spastic form of cerebral palsy (89.7%, *n* = 61 for group A, and 81.8%, *n* = 54 for group B), while the topography founded most frequently were those with bilateral involvement. The EDACS scale allows to evaluate the safety and efficacy of swallowing, being level I the one with better efficacy and safety and level V with greater difficulty of swallow and risk of aspiration. A comparative analysis was performed by group and between the initial and final EDACS classification, finding a significant difference between groups (*p* < 0.001) and where we observed that in general those that showed the greatest improvement in the level of EDACS, especially in the toxin application group were those with initial levels of EDACS IV and V, as shown in Table 2.

**Table 1:** Frequencies and means by treatment groups according to age, and characteristics of cerebral palsy.

**Variable**	**Group A** **(exclusive oromotor therapy)** **50.7% (*n* = 68)**	**Group B** **(oromotor therapy + botulinum toxin therapy)** **49.3% (*n* = 66)**
**Sex**		
Female	33.8% (*n* = 23)	33.3% (*n* = 22)
Male	66.2% (*n* = 45)	66.7% (*n* = 44)
**Age**		
1–3 years	10.3% (*n* = 7)	18.2% (*n* = 12)
4 a 6 years	38.2% (*n* = 26)	40.9% (*n* = 27)
7 a 9 years	20.6% (*n* = 14)	18.2% (*n* = 12)
10 a 12 years	14.7% (*n* = 10)	15.2% (*n* = 10)
13+ years	16.2% (*n* = 11)	7.6% (*n* = 5)
**Predominant motor disorder**		
Spastic	89.7% (*n* = 61)	81.8% (*n* = 54)
Ataxic	1.5% (*n* = 1)	0% (*n* = 0)
Disquinetic	5.9% (*n* = 4)	15.2% (*n* = 10)
Mixed	2.9% (*n* = 2)	3% (*n* = 2)
**CP topography**		
Hemiparesis	7.4% (*n* = 5)	37.9% (*n* = 25)
Diparesis	39.7% (*n* = 27)	15.2% (*n* = 10)
Double-Hemi	22.1% (*n* = 15)	34.9% (*n* = 23)
Cuadriparesis	30.9% (*n* = 21)	12.1% (*n* = 8)
**GMFCS**		
I	35.3% (*n* = 24)	19.7% (*n* = 13)
II	19.1% (*n* = 13)	7.6% (*n* = 5)
III	14.7% (*n* = 10)	7.6% (*n* = 5)
IV	25% (*n* = 17)	34.8% (*n* = 23)
V	5.9% (*n* = 4)	30.3% (*n* = 20)

**Table 2:** Swallowing efficacy and safety levels before and after evaluation by treatment group (*p* < 0.001).

**Treatment group**			**EDACS (initial)**					**Total**
			**I**	**II**	**III**	**IV**	**V**	
Group A (exclusive oromotor therapy)	EDACS (final)	I	30					30
		II		21	1			22
		III			10	1		11
		IV				3	1	4
		V					1	1
	Total		30	21	11	4	2	68
Group B (oromotor therapy + botulinum toxin therapy)	EDACS (final)	I	16					16
		II		18				18
		III			17	4		21
		IV				9	1	10
		V					1	1
	Total		16	18	17	13	2	66
Total	EDACS (final)	I	46					46
		II		39	1			40
		III			27	5		32
		IV				12	2	14
		V					2	2
	Total		46	39	28	17	4	134

The Bruni Childhood Sleep Disorder Scale (SDSC) was applied. Comparison of means for independent samples with student's *T* for both treatment groups for initial and final measurement was performed, finding the means of each subscale and their confidence intervals, as well as the significance (Table 3).

**Table 3:** SDSC means and confidence intervals (initial and final by treatment group).

**SDSC**	**Group A** **(exclusive oromotor therapy)** **50.7% (*****n*** **=** **68)**			**Group B** **(oromotor therapy** **+** **botulinum toxin therapy)** **49.3% (*****n*** **=** **66)**		
	**Initial**	**Final**	***p***	**Initial**	**Final**	***p***
Disorders of initiating and maintaining sleep	9.79 (IC95% 9.08–10.54)	9.47 (IC95% 8.86–10.11)	0.40	10.86 (IC95% 10.04–11.77)	9.86 (IC95% 9.27–10.54)	0.07
Sleep breathing disorders	4.64 (IC95% 4.04–5.26)	4.10 (IC95% 3.60–4.60)	0.11	6.74 (IC95% 5.89–7.51)	4.75 (IC95% 4.12–5.36)	<0.01
Disorders of arousal	3.50 (IC95% 3.30–3.70)	3.41 (IC95% 3.23–3.60)	0.18	3.95 (IC95% 3.68–4.25)	3.77 (IC95% 3.54–4.03)	0.12
Sleep-wake transition disorders	2.94 (IC95% 1.83–3.92)	7.23 (IC95% 6.66–7.83)	0.20	8.33 (IC95% 7.54–9.25)	7.84 (IC95% 7.21–8.57)	0.13
Disorders of excessive somnolence	5.80 (IC95% 5.45–6.20)	5.67 (IC95% 5.35–6.02)	0.29	6.15 (IC95% 5.78–6.56)	5.92 (IC95% 5.60–6.24)	0.19
Sleep hyperhydrosis	2.29 (IC95% 2.11–2.47)	2.23 (IC95% 2.07–2.41)	0.01	2.72 (IC95% 2.48–3.00)	2.62 (IC95% 2.37–2.87)	0.01
TOTAL SDSC score	33.50 (IC95% 31.36–35.60)	32.13 (IC95% 30.32–33.82)	0.065	38.77 (IC95% 36.48–41.40)	34.78 (IC95% 32.96–36.78)	0.003

We observed that although both therapies result in favorable changes in the mean of all subscales of SDSC and in the total score of the same, the joint therapy of application of abobotuliniumtoxinA and orofacial therapy is of greater benefit (*p* = 0.003 and *p* = 0.06, respectively), especially with respect to Sleep Breathing Disorders and Disorders of initiating and maintaining sleep (*p* < 0.01 and *p* = 0.07, respectively). Hyperhidrosis subscale result is similar in both groups.

The SDSC has a cut-off point for sleep disorders with a score of 39, when comparing the initial and final scores we found that for group A (Exclusive Orofacial Therapy), three of the 15 cases that were identified with sleep disorder came out of this diagnosis, representing 20% of them. While group B where they received orofacial therapy and toxin application, 13 of the 30 patients who were identified with sleep disorders initially came out of this diagnosis (43.3%), with a significant difference for this comparison (*p* < 0.001; Table 4).

**Table 4:** Relation between treatment groups in the initial and final Sleep Disorders Subscales (SDSC).

	**Initial/final**	**SDSC sleep initiation and maintenance (initial/final) (** ***p*** **<** **0.001)**			**SDSC respiratory disturbance (initial/final) (** ***p*** **<** **0.001)**			**SDSC arousal disturbance (initial/final) (** ***p*** **=** **0.014)**			**SDSC sleep/awake disorder (inicial/final) (** ***p*** **=** **0.07)**			**SDSC somnolence (initial/final) (** ***p*** **=** **0.052)**			**SDSC hyperhidrosis (initial/final) (** ***p*** **<** **0.001)**		
		**No observed alteration**	**At risk (requires observation and follow up)**	**Altered (intervention is suggested)**	**No observed alteration**	**At risk (requires observation and follow up)**	**Altered (intervention is suggested)**	**No observed alteration**	**At risk (requires observation and follow up)**	**Altered (intervention is suggested)**	**No observed alteration**	**At risk (requires observation and follow up)**	**Altered (intervention is suggested)**	**No observed alteration**	**At risk (requires observation and follow up)**	**Altered (intervention is suggested)**	**No observed alteration**	**At risk (requires observation and follow up)**	**Altered (intervention is suggested)**
**Group I (exclusive oromotor therapy) 50.7% (*****n*** **=** **68)**	**At risk (requires observation and follow up)**	**0**	57	0	6^**^	46	0	1^**^	58	7	4^**^	55	1	2^**^	62	3	2^**^	66	0
	**Altered (intervention is suggested)**	**0**	2^*^	9	0	1^*^	15	0	0	2	0	0	8	0	0	1	0	0	0
**Group II (oromotor therapy** **+** **botulinum toxin therapy) 49.3% (*****n*** **=** **66)**	**At risk (requires observation and follow up)**	**0**	49	0	25^**^	4	0	0	49	5	5^**^	52	2	3^**^	63	0	2^**^	62	0
	**Altered (intervention is suggested)**	**0**	11^*^	6	0	1^*^	36	0	1^*^	11	0	0	7	0	0	0	0	0	2
**Total**	**At risk (requires observation and follow up)**	**0**	106	0	31^**^	50	0	1^**^	107	12	9^**^	107	3	5^**^	125	3	4^**^	128	0
	**Altered (intervention is suggested)**	**0**	13^*^	15	0	2^*^	51	0	1^*^	13	0	0	15	0	0	1	0	0	2
	**TOTAL**	**0**	119	15	31	52	51	1	108	25	9	107	18	5	125	4	4	128	2

* *Are those cases that improved from the category “Altered (intervention is suggested)” to “At Risk (Requires Observation and Follow Up)”*.

** *Are those cases that improved from the category “At Risk (Requires Observation and Follow Up)” to “No Observed Alteration”*.

By breaking down each of the subscales and see the risk change according to the parameters of each one of them we found that for the subscale of Disorders of initiating and maintaining sleep, 18% (*n* = 2) the patients in group A who started with alteration in this subscale, evolved to only risk after the intervention, while 64% (*n* = 11) of the patients of group B whom started with alteration in this subscale evolved to only risk (*p* > 0.011). In the case of the Sleep Breathing Disorders subscale for group A, six patients went from Risk classification to No alteration, representing 11% of the 52 that were at risk in the initial assessment. In the case of the abobotuliniumtoxinA application group, we found that 25 of the 29 patients initially at risk evolved to the “No alteration” classification, which represents 86% of them, highlighting that this is the subscale where improvement was found in both groups, but more strongly for the abobotuliniumtoxinA application group accompanied by orofacial therapy (*p* < 0.001). In the subscale of Disorders of arousal, we found that only one case of the 59 went from risk to no alteration for group A and a single case of the 108 changed from Alteration to risk for group B (*p* = 0.014; Table 4). In the subscale of sleep/wake disturbance, we found a similar behavior in both groups, for group A 6% (*n* = 4) of the 59 that were at risk passed to the group without alteration, while in the group B had this improvement 8% (*n* = 5) of the 57 that were in this subscale (*p* = 0.7). In the subscale of Disorders of excessive somnolence we found improvement in both groups in a very similar way, where two of 64 patients in group A (3%) and three of 66 patients in group B (4%) passed the risk classification to be without alteration (*p* = 0.052) Finally for the hyperhidrosis subscale, in both cases there were two patients from each treatment group who went to the classification of “without alteration,” representing 3% (*n* = 2) in each group (*p* < 0.001; Table 4).

## Discussion

Cerebral palsy is the most common motor disability worldwide, causing several alterations at different levels (9). The objective of this study was to identify the effects of the application of abobotuliniumtoxinA and oromotor therapy, compared to exclusive oromotor therapy in patients with CP, and their impact on sleep disorders, and sialorrhea, considering that they are elements that have a negative impact in the quality of life and general health of children with this diagnosis.

The prevalence of sialorrhea in patients with CP has been reported in many studies, with a wide range between 10 and 58% of patients (10). Sialorrhea was especially frequent in the bilateral varieties (Diparesia, Quadriparesia, and double-hemiparesia).

There are different scales to assess eating and drinking abilities, one of them being the EDACS, considered as a qualitative scale that evaluates the safety and efficacy in swallowing (11, 12), with the advantages of being quick, non-invasive and sensitive, so its use is recommended in the routine evaluation of patients with CP.

Both the sialorrhea and swallowing disorders in varying degrees were studied in our sample and we found a directly proportional relationship between the intensity and frequency of the sialorrhea and the severity level of the GMFCS, meaning that those patients with higher degrees of sialorrhea, were more compromised in the safety and efficacy of swallowing and were those with a higher level of GMFCS. In their 2004 study, Senner and collaborators indicated that GMFCS scores did not correlate significantly with severity of sialorrhea, but did find an association with swallowing disorders however, their sample was smaller than that of our study (13).

Sleep disorders are frequent in the population with CP, authors like Lélis (14) have described the possible correlation of extrinsic and intrinsic factors that modify respiratory patterns in children with CP, including motor limiting factors, spasticity and the presence of involuntary movements, furthermore, in his 2016 study, she refers to the possible relationship of these disorders with dysphagia and salivary incontinence. We found that those with greater severity of sialorrhea had in fact, a greater severity of sleep disorders, especially those related to Disorders of initiating and maintaining sleep, sleep breathing disorders and sleep-wake transition disorders. We can infer that the difficulty of breathing has to do with the increase of secretions in the mouth and upper respiratory tract, which restricts the entry of air and causes frequent awakenings, generating a vicious circle between fatigue, postural alteration, spasticity, favoring alterations in sleep, and so on (14–16). Therefore, early detection and timely treatment of hypersalivation is a factor that can potentially have a positive impact on sleep and as a coadjuvant factor to reduce motor disorders in patients with CP.

When we evaluated the impact of oromotor therapy and toxin in conjunction with traditional therapy, we observed that both had a positive impact on the control of the sialorrhea, however the joint therapy of toxin and oromotor stimulation obtained better results in practically all the variables, from the safety of swallowing (Table 3 of results), to the subscales of sleep disorders, especially in those patients with more severe cases of sialorrhea and motor disorders, were those more benefited from the joint management of traditional oromotor therapy and application of abobotuliniumtoxinA, which had been documented by authors such as Savarese (17), Fuster Torres (18), Alrefai (19), Wu (20), Fitzgerald (15), and Lakraj (21).

None of the studies mentioned above evaluated the effect of swallowing on sleep disorders as in our study, where we used the SDSC, a highly useful clinical scale, of rapid application that allows identifying possible sleep disturbances and their different subscales and where we found that all the subscales of both groups presented improvement in SDSC scores, however the results of the therapy were better Orofacial along with the application of botulinum toxin to salivary glands as a way to enhance each other, the toxin decreases the amount of secretion and the oromotor therapy favors the swallowing of it, especially with a beneficial effect on the subscales of sleep breathing disorders and disorders of initiating and maintaining sleep, as well as the average SDSC total score.

The realization of orofacial therapy in patients with sialorrhea and sleep disorders exclusively is useful, however it can be enhanced with the application of abobotuliniumtoxinA at salivary glands, in cases that require it, so we suggest that always that it is feasible to combine both therapies will have a greater impact in the reduction of complications, in sleep disorders and finally in the quality of life and prognosis of patients with CP, especially in those cases with greater motor commitment. We should consider that the EDACS scale is a qualitative scale of efficacy and safety, so in general discrete modifications can be expected in the short term, we consider convenient that these patients have a follow-up to be able to observe greater changes.

In this sample, no major adverse effects were found, only those expected from the application of the toxin, such as pain, mild, and transient local inflammation.

### Study Limitations

The most important limitation of the study was the difficulty to execute quantitative studies such as polysomnography and FEES for all the patients. In this regard, work is being done to monitor these tools in future studies. Another limitation was the sample size which could be bigger what could make possible to paired participants by GMFCS. A third limitation is that we used subjective scales although they are sensitive and specific, being qualitative can cause underdiagnosis. In the follow-up of this line of research, work will be done so that in later studies randomized studies are carried out to improve their quality.

## Conclusions

There is a relation between the greater frequency and intensity of the sialorrhea in the cases of greater severity of the GMFCS of CP, also related with the frequency of sleep disorders and dysphagia.

Conventional orofacial therapy (oral cavity management, swallowing stimulation, oral sensory techniques, etc.) proved useful for improving the safety and efficacy of swallowing, however it was overcome by the mixed therapy of orofacial therapy and application of abobotuliniumtoxinA, with significant statistical difference, with changes especially in the degrees of greatest severity of swallowing.

The realization of exclusive orofacial therapy in patients with sialorrhea and sleep disorders is useful, however, it can be enhanced with the application of abobotuliniumtoxinA at salivary glands.

All subscales of both groups presented improvement in SDSC scores, however, the results of orofacial therapy were better together with the application of abobotuliniumtoxinA to salivary glands, especially for the subscales of sleep breathing disorders and disorders of initiating and maintaining sleep, as well as the average SDSC total score.

We suggest the systematic use of tools for the identification of sleep disorders, swallowing efficacy, and safety and sialorrhea in patients diagnosed with CP, the integral treatment of these clinical elements, as well as their channeling to other specialties when necessary.

## Data Availability Statement

The raw data supporting the conclusions of this article will be made available by the authors, without undue reservation.

## Ethics Statement

The studies involving human participants were reviewed and approved by Comité de Bioética e Investigación del INR. Written informed consent to participate in this study was provided by the participants' legal guardian/next of kin.

## Author Contributions

All the authors actively participated in the development of this research protocol, in the case of the corresponding author he was tutor, guide and responsible for all questions of analysis and integration of the protocol and the results. MA-S is the clinical manager of the area where the intervention was carried out, and under her guidance. KR-M and JM-V carried out the evaluations and procedures for their subsequent integration into the database and their analysis.

## Funding

The first author of this research project received a doctoral scholarship from the National Council of Science and Technology of Mexico (CONACyT), as he belongs to the Master's and Doctorate Program in Medical, Dental and Health Sciences with the number from CVU253582.

## Conflict of Interest

The authors declare that the research was conducted in the absence of any commercial or financial relationships that could be construed as a potential conflict of interest.

## Publisher's Note

All claims expressed in this article are solely those of the authors and do not necessarily represent those of their affiliated organizations, or those of the publisher, the editors and the reviewers. Any product that may be evaluated in this article, or claim that may be made by its manufacturer, is not guaranteed or endorsed by the publisher.

## Abbreviations

CP: cerebral palsy
INR-LGII: Instituto Nacional de Rehabilitación Luis Guillermo Ibarra Ibarra, México
EOMT: exclusive oromotor therapy
JT: joint therapy (abobotuliniumtoxinA in salivary glands and oromotor therapy)
DSFS: Drooling severity and frequency Scale
GMFCS: Gross Motor Function Classification System
SDSC: Sleep disturbance Scale for Children
EDACS: Eating and Drinking Ability Classification System.

## References

[1] Rosenbaum P Paneth N Leviton A Goldstein M Bax M Damiano D. A report: the definition and classification of cerebral palsy April 2006 [published correction appears in Dev Med Child Neurol. 2007;49:480]. Dev Med Child Neurol Suppl. (2007) 109:8–14. 10.1111/j.1469-8749.2007.tb12610.x 17370477

[2] Petersen S Francis KL Reddihough DS Lima S Harvey A Newall F. Sleep problems and solution seeking for children with cerebral palsy and their parents. J Paediatr Child Health. (2020) 56:1108–13. 10.1111/jpc.14830 32100418

[3] Mier RJ Bachrach SJ Lakin RC Barker T Childs J Moran M. Treatment of sialorrhea with glycopyrrolate: a double-blind, dose-ranging study. Arch Pediatr Adolesc Med. (2000) 154:1214–18. 10.1001/archpedi.154.12.1214 11115305

[4] Weitzman RE Kawai K Nuss R Hughes A. A 10-year retrospective review of botulinum toxin injections and surgical management of sialorrhea. Cureus. (2020) 12:e7916. 10.7759/cureus.7916 32494530 PMC7263709

[5] Daniel SJ Cardona I. Onabotulinum toxin A for the treatment of sialorrhea in familial dysautonomia. Int J Pediatr Otorhinolaryngol. (2014) 78:879–81. 10.1016/j.ijporl.2014.02.011 24725647

[6] Duarte CS Alves TR Azeredo PM Almeida SI. Tratamiento de la sialorrea con toxina botulínica tipo A en la parálisis cerebral. Rev Neurol. (2009) 49:610. 10.33588/rn.4911.2008103 19921629

[7] Porte M Chaléat-Valayer E Patte K D'Anjou MC Boulay C Laffont I. Relevance of intraglandular injections of botulinum toxin for the treatment of sialorrhea in children with cerebral palsy: a review. Eur J Paediatr Neurol. (2014) 18:649–57. 10.1016/j.ejpn.2014.05.007 24931915

[8] Pal PK Calne DB Calne S Tsui JKC. Botulinum toxin A as treatment for Drooling saliva in PD. Neurology. (2000) 54:244–7. 10.1212/WNL.54.1.244 10636161

[9] Stavsky M Mor O Mastrolia SA Greenbaum S Than NG Erez O. Cerebral palsy-trends in epidemiology and recent development in prenatal mechanisms of disease, treatment, and prevention. Front Pediatr. (2017) 5:1–10. 10.3389/fped.2017.00021 28243583 PMC5304407

[10] Dias BLS Fernandes AR Maia Filho H de S. Sialorreia em crianças com paralisia cerebral. J Pediatr. (2016) 92:549–58. 10.1016/j.jped.2016.03.006 27281791

[11] Van Hulst K Snik DAC Jongerius PH Sellers D Erasmus CE Geurts ACH. Reliability, construct validity and usability of the Eating and Drinking Ability Classification System (EDACS) among Dutch children with cerebral palsy. J Pediatr Rehabil Med. (2018) 11:115–24. 10.3233/PRM-170515 30010151

[12] Sellers D Bryant E Hunter A Campbell V Morris C. The eating and drinking ability classification system for cerebral palsy: a study of reliability and stability over time. J Pediatr Rehabil Med. (2019) 12:123–31. 10.3233/PRM-180581 31227668

[13] Senner JE Logemann J Zecker S Gaebler-Spira D. Drooling, saliva production, and swallowing in cerebral palsy. Dev Med Child Neurol. (2004) 46:801–6. 10.1111/j.1469-8749.2004.tb00444.x 15581152

[14] Lélis ALPA Cardoso MVLM Hall WA. Sleep disorders in children with cerebral palsy: an integrative review. Sleep Med Rev. (2016) 30:63–71. 10.1016/j.smrv.2015.11.008 26874066

[15] Fitzgerald DA Follett J Van Asperen PP. Assessing and managing lung disease and sleep disordered breathing in children with cerebral palsy. Paediatr Respir Rev. (2009) 10:18–24. 10.1016/j.prrv.2008.10.003 19203740

[16] Hartzell LD Guillory RM Munson PD Dunham AK Bower CM Richter GT. Tongue base suspension in children with cerebral palsy and obstructive sleep apnea. Int J Pediatr Otorhinolaryngol. (2013) 77:534–7. 10.1016/j.ijporl.2013.01.001 23357781

[17] Savarese R Diamond M Elovic E Millis SR. Intraparotid injection of botulinum toxin a as a treatment to control sialorrhea in children with cerebral palsy. Am J Phys Med Rehabil. (2004) 83:304–13. 10.1097/01.PHM.0000104680.28335.B9 15024333

[18] Fuster Torres MA Berini Aytés L Gay Escoda C. Salivary gland application of botulinum toxin for the treatment of sialorrhea. Med Oral Patol Oral Cir Bucal. (2007) 12:511–7. 17978775

[19] Alrefai AH Aburahma SK Khader YS. Treatment of sialorrhea in children with cerebral palsy: a double-blind placebo-controlled trial. Clin Neurol Neurosurg. (2009) 111:79–82. 10.1016/j.clineuro.2008.09.001 18977585

[20] Wu KPH Ke JY Chen CY Chen CL Chou MY Pei YC. Botulinum toxin type A on oral health in treating sialorrhea in children with cerebral palsy: a randomized, double-blind, placebo-controlled study. J Child Neurol. (2011) 26:838–43. 10.1177/0883073810395391 21551374

[21] Lakraj AA Moghimi N Jabbari B. Sialorrhea: anatomy, pathophysiology, and treatment with emphasis on the role of botulinum toxins. Toxins. (2013) 5:1010–31. 10.3390/toxins5051010 23698357 PMC3709276

